# Protamine Sulfate Is a Potent Inhibitor of Human Papillomavirus Infection *In Vitro* and *In Vivo*

**DOI:** 10.1128/AAC.01513-21

**Published:** 2022-01-18

**Authors:** Jesse M. Young, Amira Zine El Abidine, Ricardo A. Gómez-Martinez, Virginie Bondu, Rosa T. Sterk, Zurab Surviladze, Michelle A. Ozbun

**Affiliations:** a Department of Molecular Genetics & Microbiology, The University of New Mexico School of Medicine, Albuquerque, New Mexico, USA; b Department of Obstetrics & Gynecology, The University of New Mexico School of Medicine, Albuquerque, New Mexico, USA; c The University of New Mexico Comprehensive Cancer Center, Albuquerque, New Mexico, USA

**Keywords:** antiviral agents, heparan sulfate, intraepithelial neoplasm, microbicide, tumors, warts

## Abstract

Human papillomavirus (HPV) infections are transmitted through sexual or other close contact and are etiologically associated with epithelial warts, papillomas, and intraepithelial lesions that may progress to cancer. Indeed, 4.8% of the global cancer burden is linked to HPV infection. Highly effective vaccines protect against two to nine of the most medically important HPV genotypes, yet vaccine uptake is inadequate and/or cost prohibitive in many settings. With HPV-related cancer incidence expected to rise over the coming decades, there is a need for effective HPV microbicides. Herein, we demonstrate the strong inhibitory activity of the heparin-neutralizing drug protamine sulfate (PS) against HPV infection. Pretreatment of cells with PS greatly reduced infection, regardless of HPV genotype or virus source. Vaginal application of PS prevented infection of the murine genital tract by HPV pseudovirions. Time-of-addition assays where PS was added to cells before infection, during infection, or after viral attachment demonstrated strong inhibitory activities on early infection steps. No effect on virus infection was found for cell lines deficient in heparan sulfate expression, suggesting that PS binds to heparan sulfate on the cell surface. Consistent with this, prophylactic PS exposure prevented viral attachment, including under low-pH conditions akin to the human vaginal tract. Our findings suggest PS acts dually to prevent HPV infection: prophylactic treatment prevents HPV attachment to host cells, and postattachment administration alters viral entry. Clinical trials are warranted to determine whether protamine-based products are effective as topical microbicides against genital HPVs.

## INTRODUCTION

Human papillomaviruses (HPVs) are a group of highly transmissible, nonenveloped, double-stranded DNA viruses that display a tropism for cutaneous or mucosal epithelia. Of the currently recognized >200 distinct HPV genotypes (1), ≈40 HPV genotypes in the alpha human papillomavirus (α-HPV) genus cause infections in the anogenital and oral mucosa (reviewed in reference 2). Sexual transmission of these HPVs is very common, with an estimated 80% risk of infection before age 45 years (3). Infections may manifest as genital warts (condyloma acuminata), respiratory papillomatosis, and intraepithelial lesions. Virus genotypes can be grouped based on their propensity to cause lesions that may progress to cancer (2). “Low-risk” α-HPVs (e.g., genotypes 6 and 11) rarely cause cancer, but significant morbidities such as genital warts and recurrent respiratory papillomatosis (RRP) can occur (4). Infections caused by “high-risk” α-HPVs (e.g., genotypes 16, 18, and 31) have an increased likelihood of cancer progression. Moreover, the β−HPVs (e.g., genotypes 5 and 8) are associated with nonmelanoma skin cancers in certain immunocompromised individuals (5). Importantly, nearly 5% of the global cancer burden can be attributed to HPV infections (6).

Efficacious HPV vaccines have reduced the burden of HPV-related genital diseases and juvenile RRP primarily in high-income countries (7). However, HPV immunization programs in low- and middle-income countries, where the need is greatest, have poorer coverage, with the HPV vaccine worldwide reaching less than 15% of young women (8). Even in the United States, vaccine uptake for 13- to 17-year-olds is estimated to be only 54.2% (9). Thus, the incidence of HPV-related cancers is expected to increase through the year 2040 (https://gco.iarc.fr/tomorrow/en). Beyond vaccination, there are limited means of inhibiting HPV infections *in vivo*, no effective antiviral HPV therapies, and no cure for HPV infections. These issues highlight the need to better understand the process of HPV infection and identify additional preventative approaches.

To initiate an infection, HPV virions must bind to the host cell and activate signaling for receptor complex recruitment, endocytosis, and proper intracellular trafficking for genome delivery to the cell nucleus (10). HPV attachment is mediated predominantly by heparan sulfate (HS) polysaccharide chains (11–14). HS polymers are covalently linked to proteoglycan cores expressed on the plasma membrane that attach to laminin 322 (LN332) within the extracellular matrix (ECM) of human keratinocytes (HKs) (15). Heparan sulfonated proteoglycans (HSPGs) can be enzymatically cleaved and released as HS-containing proteoglycan ectodomains, and HS molecules can be cleaved by heparanase, allowing HSPG and HS to accumulate in the ECM (15); subsequently, HPV can interact with HS present at the membrane or within the ECM (13, 14). Experimentation with heparin, a structurally related polysaccharide with higher N- and O-sulfation than HS, has greatly enhanced the understanding of specific HPV capsid interactions with HS (11, 16). Cryoelectron microscopy and mutational studies indicate that heparin predominantly interacts with clefts between pentavalent capsomers of L1, the major capsid protein (17–19). This interaction is mediated by net positive charges of the HPV L1 capsid proteins that bind to the negative sulfonations present in the HS side chains of HSPGs (18, 20, 21). Cell signaling induced by HPV exposure promotes assembly of tetraspanin-enriched microdomain (TEM) platforms consisting of CD151, CD63, integrins, annexin A2 tetramers, and epidermal growth factor receptor (EGFR) to facilitate virion endocytosis (22–29).

As no single cellular receptor is known to be responsible for HPV internalization, infection-blocking strategies have focused on the initial attachment to HSPGs. Two related inhibitory tactics have primarily employed laboratory-derived HPV pseudovirions (PsVs), a surrogate for HPV infection *in vitro* and in preclinical *in vivo* models (30, 31). First, carrageenan, a sulfonic polysaccharide extracted from red algae, was found to interact with the HPV capsid in a manner like HS and heparin. *In vitro*, carrageenan appeared to alter PsV attachment to cells and also to exert a postattachment inhibitory effect on infection (32). Carrageenan effectively blocked HPV PsV infection *in vitro* and in the murine cervicovaginal challenge model *in vivo* (31, 32), but was less effective in preventing HPV PsV infection in a rhesus macaque cervicovaginal model (33). However, another study reported that crude HPV preparations from organotypic (raft) epithelial tissues were differentially susceptible to carrageenan, with HPV types 18 and 31 susceptible and types 16 and 45 resistant to carrageenan’s inhibitory effect on infection *in vitro* (34). Second, and in contrast to carrageenan’s interaction with the capsid, molecules that interact with cellular HS moieties have been shown to prevent HPV PsV binding and infection *in vitro*. The agmatine-containing poly(amidoamine) polymer AGMA1, polyethylenimine (PEI), and *N*-*N*′-(bis-5 nitropyrimidyl)dispirotripiperazine derivate 27 (DSTP27) prevent the binding of HPV PsVs and other viruses that require HS for attachment (35–38). Addition of these drugs after HPV PsV attachment to cells also prevented infection (35, 37, 38), but apparently by distinct mechanisms. DSTP27 appeared to promote nonproductive endocytosis, as detected by disappearance of capsids from the cell surface by flow cytometry (35), whereas PEI was found to displace PsVs from the surface of cells (38).

None of the aforementioned compounds is FDA approved for drug use in humans. A few clinical studies show carrageenan-based formulations to be safe and effective in reducing natural HPV infection up to 38% in women when used as directed (reviewed in reference 39). Despite these positive results, other recent clinical trials investigating carrageenan’s ability to prevent HPV infection have either been withdrawn or terminated due to lack of effect and/or negative outcomes that outweighed potential prevention of new HPV infections (ClinicalTrials registration no. NCT02354144 and NCT02382419). As DSTP27 has a low metabolic stability and releases nitric oxide *in vivo*, no preclinical or clinical extensions of this agent have been reported. However, other related diazadispiroalkane derivatives show promise *in vitro* for inhibiting infection by other pathogens that use HS for attachment (40). PEI, as a gene transfer agent, has been investigated clinically by intratumoral injection in pancreatic adenocarcinoma therapy and metastatic solid tumors (ClinicalTrials registration no. NCT01274455 and NCT03739138). To our knowledge, AGMA1 has not been evaluated for clinical safety.

Based on the findings that carrageenan, AGMA1, PEI, and DSTP27 suppress HPV infection by preventing viral capsid interaction with HS, we investigated protamine sulfate (PS), a naturally occurring polypeptide originally derived from salmon sperm which forms a stable complex with heparin (41). Native protamine (molecular weight [MW], 4.1 to 5.1 kDa) is a cationic (pI 11 to 13) peptide, with arginine comprising 50 to 80% of its amino acid structure (42). PS is FDA approved for clinical use as an intravenous drug to reverse the anticoagulant activity of heparin. PS was shown to reduce infection of hepatitis B virus by 81% via interaction with HS on cells (43); however, a cursory experiment testing PS against xenograft tissue-derived HPV11 in HKs showed only minimal inhibition of infection (13). Nevertheless, based on the ability of PS to interact with heparin and ionically similar heparan sulfate chains (44–46), we hypothesized that PS could inhibit HPV binding to host human keratinocytes and thus effectively prevent infection. Herein, we show that PS is highly effective against infection by recombinant HPV PsV and quasivirions (QVs), as well as HPV virions derived from organotypic (raft) epithelial and xenograft tissues. Our data show that PS is a drug capable of altering HPV infection prior to and immediately after HPV attachment and that PS efficiently reduces HPV PsV infection in a preclinical cervicovaginal challenge model *in vivo.* These data position PS to be clinically tested for prevention of HPV infections in humans.

## RESULTS

### Protamine sulfate reduces HPV PsV infection *in vitro* agnostic to HPV genotype.

**FIG 1:**
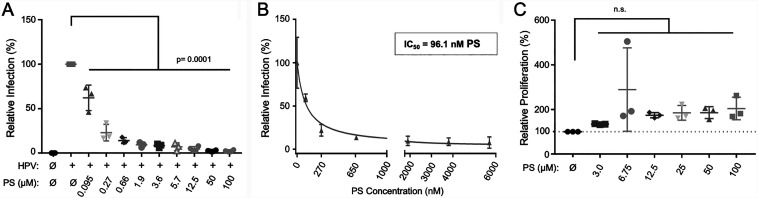
Pretreatment of HKs with protamine sulfate (PS) reduces HPV16 PsV infection. HaCaT cells were treated for 1 h with increasing concentrations of PS. (A) Thereafter, cells were exposed to HPV16 PsVs at 100 VGE/cell, and infection was assessed by luciferase assay at 48 h post-virus exposure (*n* = 3 independent experiments). Data were normalized to infected untreated cells, which were set to 100%. (B) Nonlinear regression of a dose-response curve for PS against HPV16 PsV infection; 50% inhibitory concentrations (IC_50_s) were calculated with GraphPad Prism from 3 independent experiments. (C) HaCaT cells were treated with increasing concentrations of PS for 24 h and then assessed for proliferation by CyQuant fluorescent bromodeoxyuridine (BrdU) assay. Data represent 3 independent experiments normalized to untreated cells analyzed by one-way ANOVA with multiple comparisons: *P* ≤ 0.05 for significance and n.s. for not significant.

Prior to experimentation, all virus stocks were characterized for virus capsid content, pseudogenome (via a luciferase expression plasmid) or HPV genome levels (viral genome equivalents [VGE]), and susceptibility to neutralization by characterized monoclonal antibodies as we previously detailed (47) (see Fig. S1 in the supplemental material). We first assessed the ability of PS to reduce infection using HPV PsVs encapsidating a luciferase reporter plasmid and representing HPV16, the most common oncogenic HPV genotype (48). HaCaT human keratinocyte (HK) cells were treated with increasing concentrations of PS for 1 h at 37°C prior to incubation with HPV16 PsVs (100 VGE per cell) for 24 h at 37°C; PS and virus inoculum remained with the cells for the duration of the experiment. Luciferase levels were quantified 24 h post-virus exposure and normalized to infection levels in untreated cells, as reported previously (25). HPV16 PsV infection was potently inhibited by PS with a 50% inhibitory concentration (IC_50_) value of ≈96.0 nM (Fig. 1A and B). Cellular proliferation analyses revealed that PS at concentrations up to 100 μM (≈500 μg/mL) did not alter HK proliferation (Fig. 1C). These results suggest that PS inhibits HPV PsV infection in HaCaT cells independent of influencing cellular proliferation.

**FIG 2:**
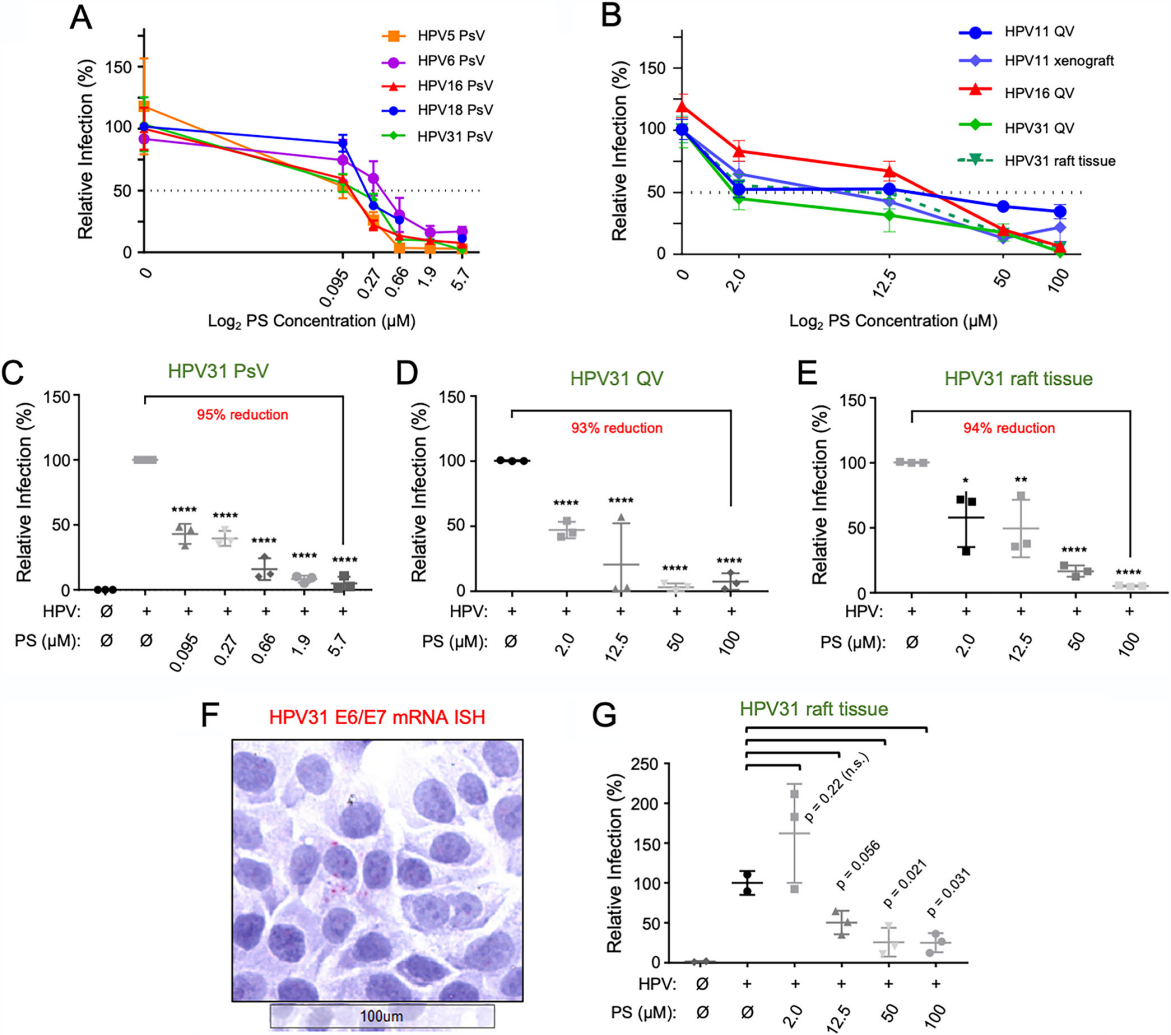
Protamine sulfate reduces infection of recombinant HPVs (PsVs and QVs) and tissue-derived HPV virions. As in Fig. 1, HaCaT cells were treated with PS for 1 h prior to exposure with HPV virions for 24 to 48 h. (A) Cells were exposed to HPV PsV genotypes 5, 6, 16, 18, and 31; cell lysates were assayed at 24 h postinfection for luciferase expression normalized to total protein concentration as a measure of infection (*n* = 3 independent infections). (B) HaCaT cells were exposed to HPV QV genotypes 11, 16, and 31, raft tissue-derived HPV31, or xenograft-derived HPV11 virions. Total RNAs were extracted 48 h post-virus exposure and subjected to RT-qPCR for quantification of spliced HPV E1^E4 mRNAs, normalized to cloned E1^E4 cDNA copy number controls. (C) Individual data points for HPV31 from panel A, showing PsV dose-response curve (*n* = 3 independent experiments in duplicate.) (D and E) Individual data points for HPV31 virions from panel B, showing QV dose-response curve (D) and raft tissue-derived HPV31 virion dose-response curve (E). HPV31 PsV data points represent replicate luciferase averages from 3 independent assays; QVs and raft-tissue HPV PCR data points are from 3 independent assays. Asterisks indicate significance by one-way ANOVA with multiple comparisons: *, *P* ≤ 0.05; **, *P* ≤ 0.01; ***, *P* ≤ 0.001; and ****, *P* ≤ 0.0001. (F and G) HaCaT cells seeded on chamber slides were exposed to raft-derived HPV31 at 0.25 VGE/cell. (F) RNA-ISH performed for high-risk HPV E6/E7 mRNAs reveals an HPV31-infected cell (RNA-ISH focus) surrounded by uninfected cells. (G) Quantification of RNA-ISH-positive foci at each PS concentration relative to untreated infection set as 100% for the total number of cells present in each chamber and untreated control (*n* = 2 to 3 replicates). Statistical analysis was by Welch’s *t* test with *P* ≤ 0.05 for significance.

We next assessed the efficacy of PS in preventing infection by HPV PsVs representing carcinogenic α-HPV genotypes 18 and 31, the low-risk α-HPV genotype 6, and the β-HPV genotype 5. In a similar experimental design, HaCaT cells were exposed to increasing concentrations of PS for 1 h prior to the addition of HPV PsVs at a dose of 100 VGE per cell. Overall, we observed PS IC_50_ values ranging from 0.073 μM to 0.33 μM for the various PsV capsid genotypes tested (Fig. 2A and Table 1). When considering the highest PS concentration tested against PsVs (5.7 μM), high-risk HPV PsV infection was inhibited 89% for HPV18, 90% for HPV16, and 95% for HPV31. The cutaneous β-HPV5 and low-risk α-HPV6 genotypes were inhibited by 97% and 85%, respectively, at 5.7 μM PS (Fig. 2A; see Fig. S2 in the supplemental material). It was striking to note that HPV5 PsVs were strongly susceptible to PS inhibition since carrageenan was reported to have no efficacy against HPV5 PsVs (32).

**TABLE 1:** PS IC_50_ values for PsVs, QVs, and xenograft- and raft tissue-derived HPVs in HaCaT cells

HPV genotype and source	IC_50_	
	μM	μg/mL
PsVs		
β-HPV5	0.078	0.398
α-HPV6	0.33	1.68
α-HPV16	0.096	0.490
α-HPV18	0.21	1.07
α-HPV31	0.073	0.372

QVs		
α-HPV11	0.64	3.26
α-HPV16	10.2	52.4
α-HPV31	1.71	8.67

Tissue-derived virions		
α-HPV11 xenograft	8.65	44.1
α-HPV31 raft	5.03	25.7

### Protamine sulfate suppresses infection by recombinant HPV quasivirions and tissue-derived HPV virions.

Previous studies testing molecules for their potential to prevent HPV infections have relied primarily on PsV, wherein reporter gene expression from a non-HPV promoter is a surrogate for infection (32, 35, 37, 38, 49). Therefore, we evaluated the efficacy of PS against recombinant HPV QVs that encapsidate genotype-matched viral genomes (50). We also tested PS against tissue-derived virions that were harvested from organotypic epithelial (raft) tissues and from HPV-infected xenograft tissues, where virion assembly relies on the epithelial cues and differentiation closely mirroring HPV replication *in vivo* (51, 52). In contrast to infection detection via quantification of luciferase expression from a strong promoter following PsV infection, QV and tissue-derived HPV infection assays rely on quantification of HPV early mRNAs driven by the viral promoter. HaCaT cells were incubated with increasing concentrations of PS for 1 h prior to infection with HPV11, HPV16, and HPV31 QVs, raft tissue-derived HPV31, and xenograft tissue-derived HPV11. To maximize the detection of viral mRNAs as a measure of infection, total RNAs were harvested from cells 48 h after the addition of virions. Reverse transcription coupled to quantitative PCR (RT-qPCR) was performed to quantify genotype-specific HPV spliced E1^E4 mRNA levels (53, 54). Similar to our results using PsVs, HPV infection was significantly inhibited by PS in a dose-dependent manner regardless of HPV genotype or virus source (Fig. 2B). The PS IC_50_ values obtained from each HPV source are summarized in Table 1 (additional graphed data are shown in Fig. S2) and indicate that compared to PsVs, QVs and tissue-derived virions required 50- to 60-fold-higher PS concentrations to reach 50% inhibition. We hypothesized that the presence of HS in the virus stocks might contribute to higher IC_50_ values for QVs and/or raft virions. Therefore, we tested this by performing immunoblotting (IB) for HS and the HSPG syndecan-1 (Sdc-1). This revealed that the raft tissue-derived HPV31 virion stock contained substantial HS and HSPG molecules, whereas there was no detectable HS in the CsCl gradient-isolated PsV and QV preparations (Fig. S1C). Thus, the presence of HS in the virus stocks does not appear to account for the higher IC_50_ values for HPV QVs. It is important to note that direct IC_50_ comparisons among PsV and infectious HPV stocks cannot be made as the virus purities, titration methods, and infection assays differ substantially between these virus classes. Table 2 summarizes our *in vitro* data, including IC_50_ ranges for PS against HPV PsVs, QVs, and tissue-derived virus in comparison with other ionic compounds reported to prevent HPV infection *in vitro*. These comparisons reveal that PS effectively inhibits HPV infection regardless of laboratory source.

**TABLE 2:**
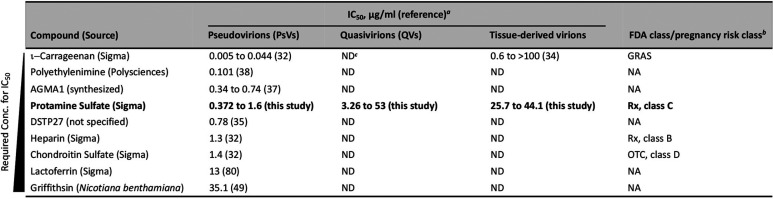
Comparison of ionic compounds shown to suppress HPV infection *in vitro*

a The values shown are based on the required concentration for the IC_50_.

b FDA class designates drug availability and separates drugs into either prescription (Rx) or over the counter (OTC) based on safety data. The pregnancy class rating refers to the safety of the drug respective to the fetus: A, no risk to fetus; B, failed to demonstrate fetal risk, but human studies are limited; C, some fetal risk, but benefit warrants use over perceived harm; D, harmful to fetus, but benefits warrant use; X, harmful and benefits do not outweigh detriment to fetus (FDA 2015 Pregnancy Guideline Amendment). GRAS, generally recognized as safe by the U.S. FDA (Food Additive Approval HEHS-94-141). NA, no FDA data or approval for use in human subjects.

c ND, not determined or no data available.

Luciferase and RT-qPCR assays rely on whole-cell lysates for quantification of relative HPV infections. Thus, these assays cannot discern whether PS treatment leads to infection of fewer cells or whether the same number of cells are infected but express lower levels of luciferase or viral mRNAs. To address this issue, we used a cell-based focus-forming assay that evaluates infection using RNA *in situ* hybridization (RNA-ISH) detection of HPV mRNA as we reported previously (47). HaCaT cells were pretreated with PS for 1 h and exposed to raft tissue-derived HPV31. Infections were quantified by counting the number of cells harboring two or more punctate RNA-ISH signals (Fig. 2F and G). Compared to untreated cells, PS reduced the total number of focus-positive cells detected by RNA-ISH. Similar to the RT-qPCR analysis showing an IC_50_ for PS against raft tissue-derived HPV31 of ≈12.5 μM, we observed an average of 50% reduction in the total number of positive cells at 12.5 μM PS and 75% reduction at 100 μM PS. Together, these findings indicate that PS significantly inhibits HPV infections regardless of HPV genotype and virus source by reducing the total number of infected cells.

### Protamine sulfate prevents HPV attachment to HKs and ECM.

**FIG 3:**
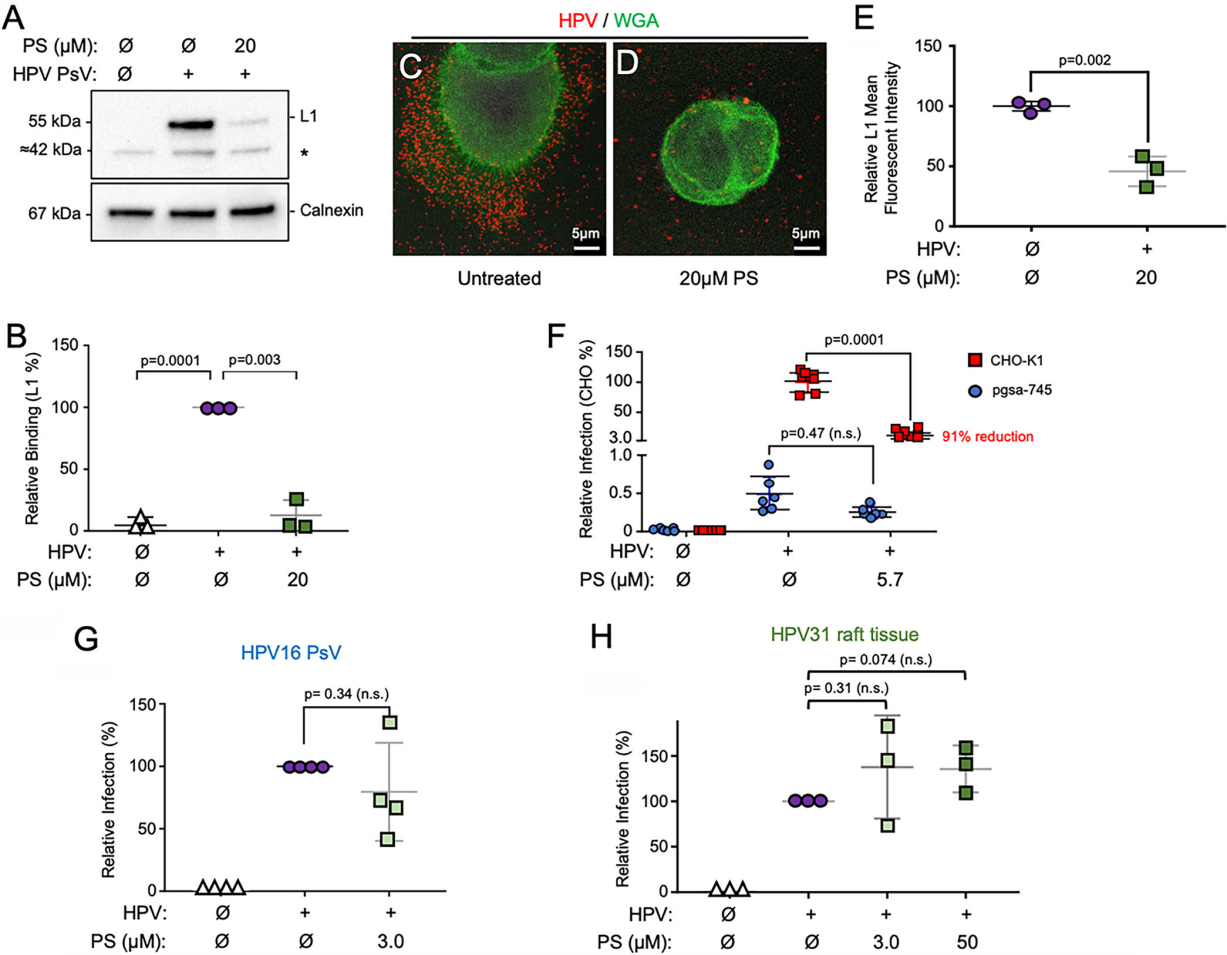
Protamine interacts with cellular HSPGs to prevent HPV binding. (A) HaCaT cell monolayers were treated with 20 μM PS (in normal HaCaT medium) before the addition of HPV16 PsVs at 4°C for 1 h. After washing to remove unbound virus particles, cells were lysed and subjected to SDS-PAGE and immunoblot detection of HPV16 major capsid protein L1 (CAMVIR-1) and cellular calnexin as a loading control. A cellular protein at ≈42 kDa is nonspecifically (*) recognized by the CAMVIR-1 antibody (58, 79). (B) Densitometry quantification of HPV L1 normalized to calnexin (*n* = 3 independent experiments) analyzed by one-way standard ANOVA. (C and D) Immunofluorescence performed on samples treated as in panel A. Cells were plated on coverslips and fixed with 4% PFA for immunofluorescence. HPV L1 (red) was stained using polyclonal anti-HPV16 VLP (1:200), and cell membranes were stained using the lectin wheat germ agglutinin (WGA; green). Representative images of untreated or PS-treated cells are shown. (E) ImageJ mean fluorescence intensity analysis of L1 signal. Each data point represents an average of three imaged areas from each independent experiment (*n* = 3). (F) Parental CHO-K1 cells or HS-null CHO cells (pgsa-745) were pretreated for 1 h with PS before being exposed to HPV16 PsVs. Infections were assessed by luciferase assay 24 h after PsV exposure (*n* = 3 independent experiments). (G) HPV16 PsVs were allowed to interact with 3 μM PS (final) in a small volume for 1 h at 37°C prior to exposure to HaCaT cells. Infection was assessed by luciferase assay 24 h post-PsV exposure. Data points represent 4 independent experiments. (H) Raft tissue-derived HPV31 virions were allowed to interact with 3 μM PS (final) or 50 μM PS (final) in a small volume for 1 h at 37°C prior to exposure to HaCaT cells. Infections were assessed by RT-qPCR assay 48 h post-HPV31 exposure. Data points represent 3 independent experiments. Student's *t* test was used for analyses in panels E to H. n.s., not significant.

Previous studies reported that PS interacts with heparin and HS molecules (44–46, 55). Therefore, we hypothesized that the ability of PS to bind to cellular HS would inhibit the interaction between HPV and HS molecules on cells and in the ECM. To test this, HaCaT cells were treated with 20 μM PS in complete medium for 1 h; cells were then exposed to HPV16 PsVs and incubated at 4°C for 1 h to permit viral attachment but prevent temperature-dependent internalization. After cells were washed to remove unbound HPV PsVs, viral binding was assessed in two ways. First, cells were extracted in radioimmunoprecipitation assay (RIPA) buffer, and lysates were analyzed by SDS-PAGE and immunoblotting for the levels of HPV16 L1 major capsid protein. This revealed that compared to untreated cells, pretreatment of cells with 20 μM PS reduced HPV16 PsV binding by an average of 88% (Fig. 3A and B), which is consistent with the reduced infection levels observed in Fig. 1A. Second, we performed the same experiment and evaluated virus binding by confocal fluorescence microscopy staining for HPV L1 capsids and the cell membrane with wheat germ agglutinin (WGA) (Fig. 3C and D). The mean fluorescence intensity of HPV L1 signals captured from three replicates each in three independent experiments showed that pretreatment of cells with 20 μM PS significantly reduced HPV binding to the ECM and the cell membrane (Fig. 3E). The lesser effect of reduced binding in this assay may reflect background fluorescence. To investigate the specific importance of HS moieties for PS-mediated inhibition of HPV infection, we compared the effects of PS on infection between HS-replete CHO-K1 cells and a CHO derivative cell line (pgsA-745) defective for xylosyltransferase activity, which results in severely reduced HS glycosaminoglycan biosynthesis (56). Whereas the CHO-K1 cells with high HS levels were readily infected by HPV16 PsVs, the HS-deficient pgsA-745 cells were poorly infected with HPV16 PsVs (Fig. 3F), as previously reported (32, 35). The pretreatment of parental CHO-K1 cells with 5.7 μM PS resulted in a 91% reduction in infection (Fig. 3F). In contrast, PsV infection of the HS-defective pgsA-745 cells was not significantly altered by PS pretreatment (Fig. 3G). Together, these data provide evidence that the ability of PS to inhibit HPV infection is dependent upon the presence of cellular HS molecules. Finally, we tested whether PS inhibited HPV infection by direct interaction with viral capsids. HPV16 PsVs and raft tissue-derived HPV31 virions were preincubated with 3 μM PS in a small volume of growth medium for 1 h at 37°C to permit virus-drug interaction. As the tissue-derived HPV31 virions required more PS to reach the IC_50_ (25.6 μM), we also incubated these virions with 50 μM PS. The PsVs and tissue virions were then diluted in medium to a final concentration of 30 nM (from 3 μM) or 500 nM (from 50 μM) PS and exposed to HaCaT cells. Preincubation of PsV with PS yielded a slight average reduction of infection, but this was not statistically significant (Fig. 3G). The minor average reduction of infection by HPV16 PsVs at a final concentration of 30 nM PS is likely due to the ability of PS to inhibit HPV infection in the low-nanomolar-concentration range, as shown in Fig. 1A. Raft tissue-derived HPV31 infection was not inhibited when the virion stock was incubated with 3.0 or 50 μM PS (Fig. 3H), despite our finding that the virion preparation contained copurifying HS molecules that might interfere with PS (Fig. S1C). Cumulatively, these results support the idea that PS primarily prevents HPV infection by blocking capsid attachment to cells and the ECM, mediated through the interaction of PS with cellular HS molecules.

### Protamine sulfate exerts postattachment inhibition of HPV infection by altering viral entry.

**FIG 4:**
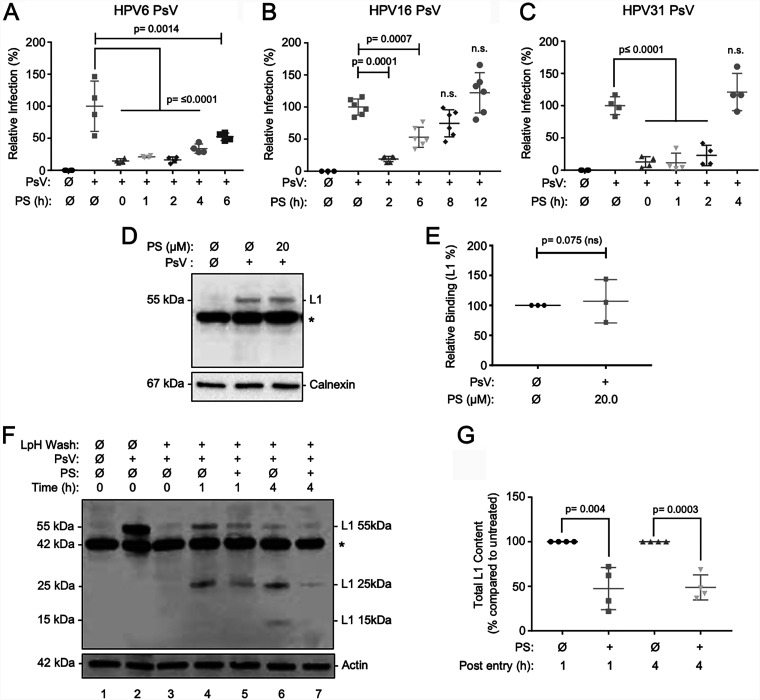
Protamine reduces infection after viral attachment by slowing entry. (A to C) HaCaT cells were exposed to HPV PsV inocula for 1 h at 4°C to allow viral attachment. Inocula were removed, and the cells were washed to remove unbound virions. Thereafter, cells were treated with PS (5.7 μM final concentration) in complete medium at the indicated times after the transition to 37°C. Infections were allowed to proceed for a total of 24 h and assessed by luciferase assay. (A) HPV6 PsVs with 2 independent experiments each in duplicate. (B) HPV16 with 3 independent experiments each in duplicate. (C) HPV31 with 2 independent experiments each in duplicate analyzed by one-way standard ANOVA. *P* ≤ 0.05 for significance. (D and E) Cells were exposed to PsVs for 1 h at 4°C. After aspiration of unbound PsVs, cells were washed, and medium containing PS (20 μM final concentration) was added for 1 h at 37°C. Cell lysates were collected in RIPA buffer and subjected to SDS-PAGE and immunoblotting for L1 (CAMVIR-1); cellular calnexin was detected as a loading control. A cellular protein at ≈42 kDa is nonspecifically recognized by the CAMVIR-1 antibody (*). Panel E graphs densitometric quantification of L1 (55 kDa) normalized to calnexin (*n* = 3 independent experiments). (F and G) HPV internalization assay wherein HPV16 PsVs were allowed to attach to HaCaT cells at 4°C for 1 h; inocula were aspirated, and cells were washed to remove unbound PsVs. Controls include cells in the absence of PsVs, where a nonspecific band at ≈42 kDa (*) is present (lane 1), total virus was bound without low-pH stripping (LpH Wash; lane 2), and removal of extracellular HPV with LpH stripping was performed to remove cell-external PsVs (lane 3). (Lanes 4 to 7) After removal of unbound PsV, media were replenished without or with PS (5.7 μM final concentration), and cells were transitioned to 37°C for 1 or 4 h prior to LpH stripping of extracellular PsVs. Cell lysates were subjected to SDS-PAGE and immunoblotting for L1; cellular actin was detected as a loading control. (G) Quantification of total L1 content (sum of 55-, 25-, and 15-kDa L1 species) normalized to actin; data for PsV-exposed cells without PS (lanes 4 and 6) were set to 100% of L1 content (*n* = 4 independent experiments). Data from immunoblots were analyzed using Student's *t* test (E and G).

Both carrageenan and DSTP27 were found to exert inhibitory effects on HPV infection after PsVs were bound to cells (32, 35). Thus, to determine the potential for PS to exert postattachment neutralization of HPV infection, PsVs were allowed to bind to HaCaT cells at 4°C for 1h. After unbound virions were removed by washing, fresh media were applied, and cells were transitioned to 37°C to allow viral entry and infection. PS was added at the time of temperature transition or at increasing time points after transition to 37°C. PS added after binding and at transition to 37°C reduced average infection by HPV16 by 81%, that by HPV6 by 86%, and that by HPV31 by 88% (Fig. 4A to C, respectively). HPV6 and HPV16 PsV infection remained significantly susceptible to PS inhibition when added up to 6 h after temperature transition (Fig. 4A and B), whereas HPV31 PsV infection was significantly suppressed when PS was added up to 2 h after transition to 37°C (Fig. 4C).

To investigate possible mechanisms whereby PS suppressed infection after HPV attachment, we first evaluated whether PS displaced virions from the cell surface. Again, PsVs were allowed to bind HaCaT cells at 4°C for 1h. After unbound virions were removed by washing, medium containing PS (20 μM final concentration) was added for 1 h at 37°C. Analyses of cell lysates by SDS-PAGE and immunoblot for HPV L1 revealed that PS exposure after virus binding did not decrease the total amount of detected L1 (Fig. 4D and E). This suggests that PS treatment does not displace the HPV virions associated with the cells. We next investigated whether PS altered viral uptake by again performing PsV binding to cells at 4°C, followed by PS treatment, and then allowing HPV entry for 0, 1, or 4 h at 37°C. To assess only internalized virions, cells were detached from the plates with trypsin-EDTA and treated with a low-pH wash to remove extracellular virus as we reported previously (14). Following cell lysis, SDS-PAGE and immunoblotting were used to determine the levels of intracellular HPV L1 protein (Fig. 4F and G). Controls without low-pH stripping showed no detection of L1 (55 kDa) in the absence of PsVs and strong detection of L1 after PsVs were bound (Fig. 4F, lanes 1 to 2, respectively). When PsVs were bound to cells without shifting to 37°C, no L1 was detected after low-pH wash, indicating that all virus was extracellular and effectively removed (Fig. 4F, lane 3). When PsV-bound cells were shifted to 37°C for 1 h, full-length L1 (55 kDa) and a 25-kDa L1 fragment were observed, indicating viral entry (Fig. 4F, lane 4): by 4 h post-HPV entry, less full-length L1 was observed, and a 15-kDa L1 fragment was also apparent (Fig. 4F, lane 6). These findings are consistent with previous reports of L1 proteolytic processing during endocytosis (57, 58). In contrast, when PS was added to virus-bound cells prior to entry initiation at 37°C, this assay revealed that HPV entry was inhibited at both 1 and 4 h post-HPV entry compared to PsV-bound cells not exposed to PS (Fig. 4F, lanes 4 to 7). Analysis of four independent experiments revealed that total L1 band intensity (the sum of the 55-, 25-, and 15-kDa species) was reduced an average of ≥50% at both 1 and 4 h post-HPV entry in the presence of PS compared to untreated controls (Fig. 4G). These results suggest that PS exposure after HPV binding acts, in part, to significantly block viral internalization and proteolytic processing. As infection was inhibited ≥81% when PS was added to HPV-bound cells, these data together suggest that a large proportion of the internalized virus population is rendered noninfectious. This is consistent with the finding that another HS-binding drug, DSTP27, also promoted noninfectious HPV entry (35).

### Protamine sulfate prevents HPV PsV infection in a preclinical cervicovaginal model *in vivo* and in the context of low vaginal pH.

**FIG 5:**
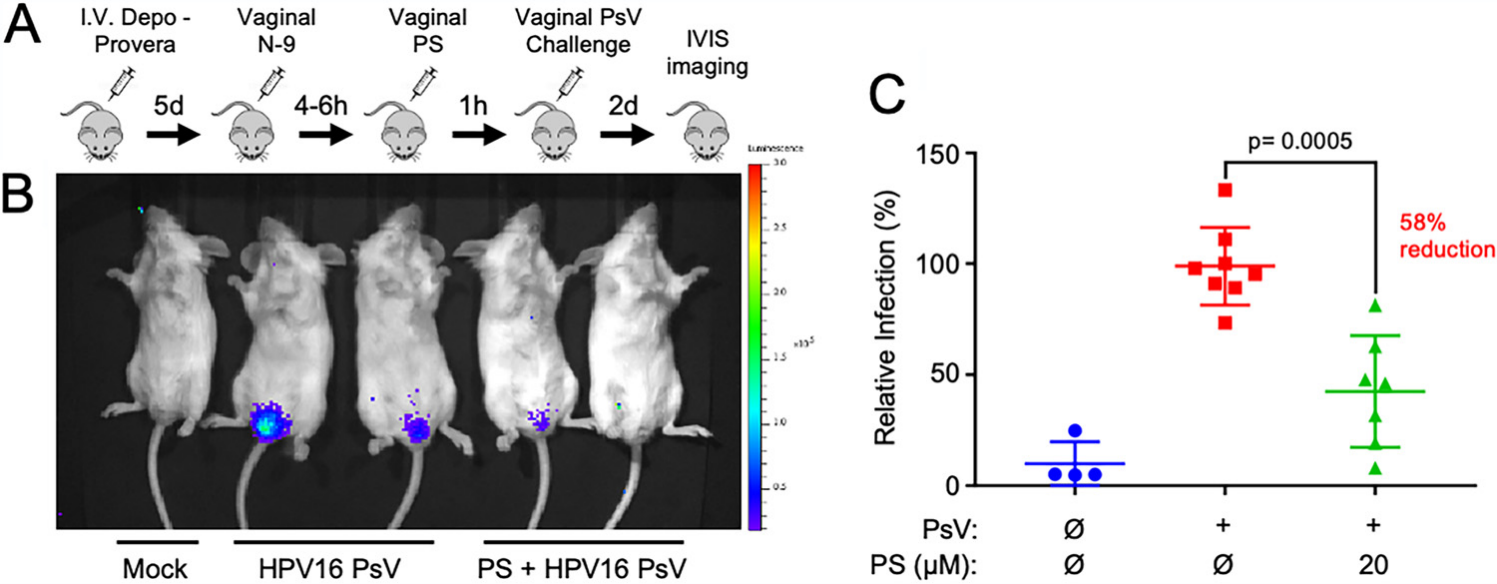
Protamine sulfate reduces infection *in vivo* in the HPV cervicovaginal challenge model. (A) Murine HPV PsV cervicovaginal challenge workflow (see Materials and Methods). (B) Representative IVIS images showing (from left to right) animals that were mock HPV exposed, HPV16 PsV exposed (positive control), and PS treated prior to HPV16 PsV exposure. (C) Quantification of luciferase signals from 3 independent experiments, including *n* = 4 mock infected, *n* = 8 HPV16 PsV exposed (experimental averages set to 100%), and *n* = 7 PS treated plus HPV16 PsV exposed relative to positive controls. Results were analyzed by Welch’s *t* test.

To investigate the effect of PS *in vivo* we utilized the mouse cervicovaginal infection model, which provides a relevant tissue context to evaluate the efficacy of drugs that may prevent the initiation of HPV infection (31, 59). Mice were prepared for intravaginal PsV instillation by progesterone-induced estrus synchronization and epithelial barrier disruption using nonoxonol-9 (N-9) treatment (Fig. 5A), according to the previously established protocol (31). At 6 h after N-9 treatment, PS was suspended in carboxymethylcellulose (CMC) to increase drug retention and vaginally instilled at a final concentration of 20 μM. HPV16 PsVs suspended in CMC were instilled 1 h later (31). Vaginal infection assessment by whole-animal bioluminescent imaging 48 h post-PsV exposure revealed that PS pretreatment significantly suppressed HPV16 infection (Fig. 5B and C). This suggests that PS can effectively reduce infection in the context of a relevant epithelial tissue model and that the murine cervicovaginal environment did not inhibit PS function.

**FIG 6:**
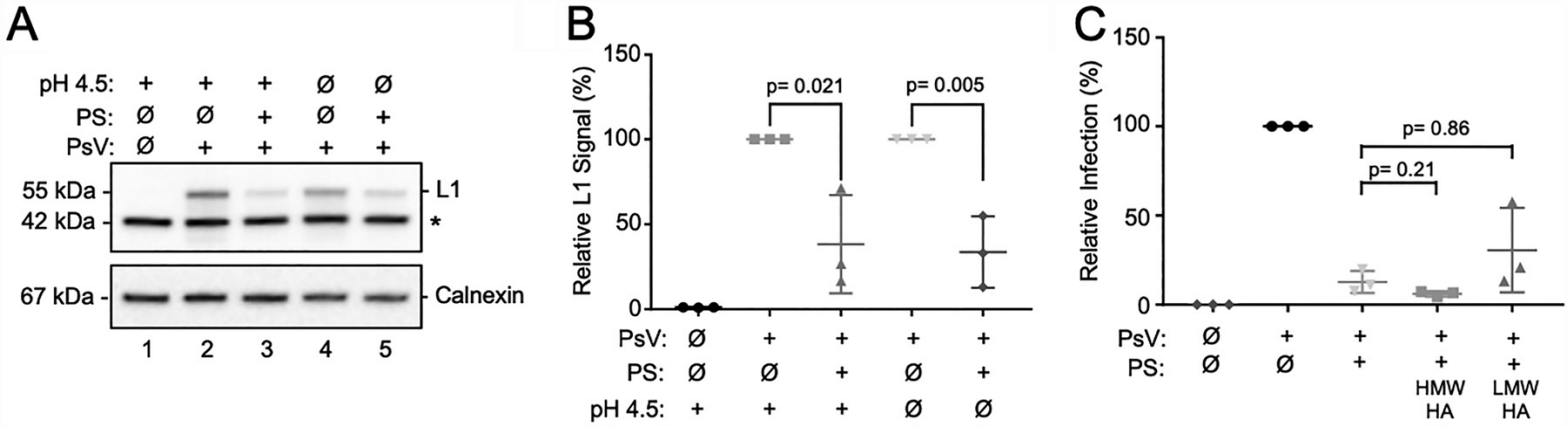
Protamine sulfate reduces HPV binding under low-pH conditions and remains functional when mixed with hyaluronic acid (HA). (A) HaCaT cells were incubated in sodium lactate buffer (pH 4.5) containing 20 μM PS for 30 min prior to HPV16 PsV attachment at 4°C for 1 h. Unbound inocula were removed by washing; cellular lysates were collected and analyzed by SDS-PAGE and IB for HPV L1 and cellular calnexin as a loading control. *, nonspecific cellular reactant. (B) Densitometric quantification of L1 (55 kDa) normalized to calnexin (*n* = 3 independent experiments) and analyzed by Student's *t* test. (C) HaCaT cells were treated with PS or PS incubated with high-molecular-weight (HMW; 1,500 to 1,800 kDa) HA or low-MW (LMW; 15 to 30 kDa) HA for 1 h at room temperature. PS-HA mixtures were used at final concentrations of 1 mg/mL HA and 3 μM PS. After treatment, cells were exposed to HPV16 PsVs, and infections were evaluated after 24 h by luciferase assay (*n* = 3 independent experiments). Results were analyzed by one-way ANOVA.

The human vaginal tract typically maintains an acidic pH, compared to the more neutral pH of mice (60, 61). To evaluate PS activity under more human-relevant conditions, we tested PS function *in vitro* at a pH of 4.5, which is considered normal for premenopausal women (61). Experiments revealed that PS maintained a similar ability to block HPV binding at pH 4.5 compared to pH 7.4 (Fig. 6A and B). Drug delivery to the vaginal cavity commonly includes muco-adhesive carrier compounds, such as hyaluronic acid (HA), cellulose derivatives, and/or pectin present in lotions and lubricants, which enhance binding and counteract the vaginal self-cleansing effects (62). Thus, we tested the effectiveness of hyaluronic acid, an anionic nonsulfonated glycosaminoglycan present in the ECM (63, 64). PS was allowed to interact with high-molecular-weight (HMW) or low-molecular-weight (LMW) hyaluronic acid at room temperature for 1 h, reaching final concentrations of 1 mg/mL HA and 3 μM PS. Cells were pretreated with PS or PS-HA mixtures for 1 h prior to exposure to HPV16 PsVs; infection was again evaluated at 24 h post-HPV exposure. Addition of either HMW or LMW hyaluronic acid to PS had no significant effect on the ability of PS to inhibit HPV infection (Fig. 6C). Overall, these results indicate that PS remains effective in suppressing HPV infection in the context of a low vaginal pH and that HA could act as an effective carrier agent for PS.

## DISCUSSION

Sexually transmitted HPV infections remain a significant problem worldwide, despite the development of effective vaccines. Herein, we show that protamine sulfate (PS), an FDA-approved drug, is a potent inhibitor of HPV infection *in vitro* and *in vivo.* The inhibitory effects of PS are agnostic to HPV genotype and infectious virion source and can function both before and after HPV exposure to host cells. Additionally, PS remains functional in the context of low pH akin to that of the human vaginal tract and suppresses infection in a preclinical murine cervicovaginal model of HPV infection. Thus, PS is an attractive candidate for use as a broad-spectrum topical microbicide to block HPV infection, potentially using hyaluronic acid, a common vaginal moisturizer, as a carrier.

PS effectively prevented infection by a spectrum of α-HPV genotypes that cause genital warts and anogenital cancers. In addition, PS efficiently blocked infection by the β-HPV5 genotype, which was striking since carrageenan was found to be unable to prevent HPV5 infection despite interaction between carrageenan and HPV5 capsids (32, 65). Of note, another study showed that PsVs from a number of other α-HPV and β-HPV genotypes were not inhibited *in vitro* by carrageenan (65). We also demonstrated that PS effectively reduced infections by recombinant HPV11, HPV16, and HPV31 quasivirions (QVs), HPV31 virions isolated from organotypic epithelial (raft) tissues, and HPV11 isolated from xenograft tissues. To our knowledge, this is the first study to compare potential HPV microbicide effects among PsV, QVs, and tissue-derived HPV virions, and xenograft-derived virions. Interestingly, our HPV31 virion stock from raft tissues contained copurifying HS molecules, which may help explain why we previously found raft-derived HPV31 could efficiently infect human keratinocytes that lacked HS molecules (54). Yet here, we showed that tissue-derived HPV31 was susceptible to PS-mediated inhibition of infection. Cruz and Meyers reported that crude HPV raft preparations were differentially susceptible to carrageenan inhibition of infection *in vitro*, with HPV16 and HPV45 resistant and HPV18 and HPV31 sensitive (34). Although no other study directly compared carrageenan’s inhibitory effects between PsVs and QVs or tissue-derived HPVs, Cruz and Meyers estimated that carrageenan was ≈1,000-fold less efficacious on crude raft virus preparations compared to effects on PsVs reported by Buck et al. (32, 34). Whereas our data suggest that PS’s IC_50_ values were higher for QVs and tissue-derived HPVs, we caution that it is unsound to make direct infectivity or IC_50_ comparisons between PsVs and infectious QVs or tissue-derived HPVs, as the virus titer characterizations, experimental designs, and infection readouts are quite distinct among these *in vitro* infection models. Nevertheless, our data herein indicate that PS effectively inhibits infections by each HPV virion stock tested, whether PsVs, recombinant QVs, or tissue derived. While laboratory-produced HPV PsVs, QVs, and tissue-derived virions and current infection models may not completely recapitulate clinical HPV infection (66), HPV PsV preclinical studies with carrageenan (31, 33) were strong predictors of the drug’s efficacy in preventing HPV acquisition in clinical trials in women (reviewed in references 39). For example, the CATCH clinical trials showed a 36% reduction in newly acquired HPV infection in women who had applied carrageenan-containing gels (67). However, there have been safety concerns and absence of protective effects with carrageenan against anal HPV infections (39, 68). Another carrageenan study (ClinicalTrials registration no. NCT02382419) was withdrawn for unknown reasons. Thus, there is a compelling need to develop improved microbicides to prevent HPV infections. Our data suggest that PS may provide broader protection than either carrageenan or currently available vaccines, which do not target β-HPV infections or many pathogenic α-HPVs.

Mechanistically, our investigations show that pretreatment of cells with PS primarily prevents HPV infection by significantly reducing binding via PS interaction with host cell HS molecules. The ability of PS to prevent HPV attachment to cells remained effective at a low pH analogous to that in the human vaginal tract. PS also suppressed infection after virions were bound to cells, similar to the effects reported for the HS-interacting drug DSTP27 on HPV infection (35). Our data indicate that the ability of PS to suppress HPV infection after virus was bound was only partially attributed to reduced viral entry, indicating that PS also renders internalized virions noninfectious. Potentially, post-HPV attachment, PS could interfere with virion transfer to the entry receptor complex, cell signaling for entry receptor platform assembly and proper endocytosis, uncoating (release) of the HPV genome-L2 complex in intracellular vesicles, and/or or intracellular trafficking of the viral genome to the nucleus. These possibilities are not mutually exclusive; however, additional studies will be needed to understand this mode of inhibition and may reveal additional insights into the cellular requirements for infectious entry of HPVs.

PS is a relatively inexpensive drug that if adapted from clinically available intravenous formulations with no refrigeration requirement, could be repurposed for wide distribution to reduce HPV transmission. Clinical exploration of PS as a topical microbicide should specifically account for known intravenous side effects, including systemic hypotension and rare anaphylactic allergic reactions (41, 69). Despite the potential side effects of intravenous administration, PS is an attractive candidate for use as a broad-spectrum topical microbicide to block HPV infection, which we show may be delivered by hyaluronic acid-based moisturizers. Further studies will be needed to determine optimal formulation and dosing of PS for clinical testing. We showed that pretreatment with PS at a final concentration of 20 μM (≈102 μg/mL) was effective in reducing HPV16 PsV infection in the murine cervicovaginal model. It is possible that substantially higher PS doses could be safely used topically based loosely on the fact that protamine is infused intravenously at 5 mg/mL and given at a rate of 5 mg (or 1 mL) per min, not to exceed 50 mg in 10 min (69). The murine papillomavirus (MmuPV1) model, wherein experimental infections can be assessed in the anal and genital tracts and can be transmitted from experimentally infected animals to their sexual partners (70, 71), should prove an important relevant preclinical model for investigating PS formulations for their initial safety, side effects, and efficacy in preventing papillomavirus infections.

In conclusion, our work shows that PS treatment is effective in preventing α- and β-HPV infections and warrants further investigations and development for clinical testing. As PS appears to prevent HPV attachment to cells via ionic interaction with cellular HS molecules, it may also inhibit infections by a broad range of sexually transmitted pathogens that require HS for infection, including human immunodeficiency virus, the herpes simplex viruses, Chlamydia trachomatis, and Neisseria gonorrhoeae (72). Overall, our data support the idea that PS should be investigated as a potential prevention strategy complementary to HPV vaccination, particularly in populations who are unable or ineligible to receive HPV vaccines.

## MATERIALS AND METHODS

### Cell lines.

Cell line sources and their culture conditions followed previous reports (25, 32, 56, 73, 74). Briefly HaCaT cells (RRID:CVCL_0038), a gift from N. Fuesnig (DKFZ), Chinese hamster ovary cells (CHO-K1) (ATCC catalog no. CCL-61), and CHO-derived pgsA-745 cells (ATCC catalog no. CRL-2242) (56) were maintained in Dulbecco’s modified Eagle’s medium (DMEM)–F-12 supplemented with 10% fetal bovine serum (FBS) (AtlantaBio, catalog no. B17015), glutamate-penicillin-streptomycin (Sigma, catalog no. G1146) and 40× amino acids (Sigma, catalog no. M5550). HEK293TT cells (75) were maintained in 1× DMEM (Gibco, catalog no. 11965-084) with 10% FBS under antibiotic-free conditions. Cell lines were checked regularly for mycoplasma by the ATCC Universal Mycoplasma Detection kit (ATCC 30-1012K), and cell line identities were validated by IDEXX short-tandem-repeat DNA profiling and multiplex PCR.

### Virus production and validation.

Recombinant HPV pseudovirions (PsVs) and quasivirions (QVs) were produced via transfection of 293TT cells as previously reported (50, 76). PsVs were engineered to encapsidate the pGL3-luciferase plasmid (Promega, catalog no. E1751) with HPV L1 and L2 capsid proteins (see Table S1 in the supplemental material); QVs were assembled with recircularized genotype-specific HPV genomes and genotype-matched HPV L1 and L2 structural proteins (Table S1) (76). Viral stocks were isolated following CsCl density gradient centrifugation and side puncture (77). When virus stock allowed, SDS-PAGE and Coomassie brilliant blue staining were used to assess purity and the presence of appropriately sized L1 (55-kDa) and L2 (72-kDa) capsid proteins and cellular histones (Fig. S1A). Virion assembly and infectivity were validated by viral neutralization assay, wherein neutralizing antibody anti-HPV16 clone H16.V5 (a gift from N. Christensen, Penn State College of Medicine) was incubated with HPV virion stocks at 37°C for 1 h prior to exposure to cells, and infection was measured 24 h after exposure. Reduction of infection by ≥99% when treated with the antibody H16.V5 was used as a metric to indicate proper capsid assembly and confirm luminescence or viral mRNA levels resulted from capsid-mediated genome delivery and not uptake of unpackaged nucleic acid, as we previously described for crude virus preparations (Fig. S1B) (47). Refined organotypic (raft) epithelial tissue-derived HPV31 virions were produced and isolated as reported previously (47). HPV11 virions from infected human keratinocyte xenografts grown in athymic mice as previously described, were a gift of Neil Christensen (Penn State College of Medicine) (52). Titers of virion stocks were physically determined for viral genome equivalents (VGE) by quantitative PCR (qPCR) as described previously (47); primers are presented in Table S2 in the supplemental material.

### HPV infections.

HaCaT human keratinocytes, CHO-K1 cells, and pgsA-745 cells were seeded at 5 × 10^4^ cells per well in 24-well plates or 1 × 10^5^ cells per well in 12-well plates to be 60 to 70% confluent overnight. Cells were exposed to doses of 0.25 to 100 VGE per cell as indicated. For prophylactic cell treatments, PS was added at concentrations ranging from 0.01 μM to 100 μM in 1 mL of complete HaCaT growth medium and incubated at 37°C for 1 h. Infections were allowed to proceed for 24 h to 120 h, depending on the experiment. Typically, PsV infections proceeded for 24 h prior to luciferase determination, whereas QV infections were incubated for 48 h to permit maximal viral mRNA transcription prior to RNA extraction and reverse transcription-qPCR (RT-qPCR) (25, 74). Unless otherwise stated, drugs and inocula remained in the culture for the duration of the assay. The specific conditions for RT-qPCR analyses were as reported previously (47), and the primers are presented in Table S3 in the supplemental material.

### Immunoblot.

HaCaT cells were plated to be subconfluent the next day at 2.5 × 10^5^ cells in 6-well plates or at 1 × 10^5^ cells on 18- by 18-mm no. 1 coverslips (VWR, catalog no. 48366 045). Cells were mock treated or PS treated (20 μM) for 1 h at 37°C and then cooled to 4°C for 15 min. Virions were added at 100 VGE/cell in 1 mL of growth medium in the bottom of the 6-well plate to cover the surface of the coverslip and allowed to attach for 1 h at 4°C with gentle rocking. For virion binding assays, inocula were aspirated, and unbound virions were removed with 1× phosphate-buffered saline (PBS) prior to solubilization with radioimmunoprecipitation assay buffer (RIPA) buffer (50 mM Tris, 150 mM NaCl, 1% Triton X-100, 0.1% SDS, 5 mM EDTA, 1% deoxycholic acid) supplemented with 1× HALT protease/phosphatase inhibitor (Pierce catalog no. 78430), and 0.2 mM sodium orthovanadate. Samples were clarified at 15,000 rpm for 10 min at 4°C and supernatants removed prior to determination of protein concentration by Bradford assay (Bio-Rad, catalog no. 5000006, Bio-Rad SmartSpec Plus). Proteins (20 μg) were analyzed by electrophoresis through precast SDS–10% polyacrylamide gels (Bio-Rad catalog no. 4561033) or SDS–4 to 20% polyacrylamide gels (Bio-Rad catalog no. 4561094). Proteins were electrophoretically transferred onto Immobilon polyvinylidend difluoride (PVDF) membranes (Bio-Rad catalog no. 1620177); membranes were blocked in 5% bovine serum albumin (BSA) and incubated with CAMVIR-1 mouse anti-HPV16 L1 monoclonal antibody (1:2,500; Santa Cruz, catalog no. sc-47699), thought to recognize amino acids 230 to 236 of L1 (78), followed by anti-mouse IgG horseradish peroxidase (HRP)-conjugated secondary antibody (1:5,000; Santa Cruz, catalog no. NA931V). Cellular actin (1:5,000; Invitrogen catalog no. MA5-11869) or cellular calnexin (1:2,500; Thermo Scientific catalog no. PA5-34665) and subsequent anti-mouse IgG secondary or HRP-conjugated anti-rabbit IgG (1:5,000; Cell Signaling, catalog no. 7074S) were detected as loading controls. Blots were imaged and proteins of interest were quantified using the Bio-Rad Chemidoc XRS+ gel imaging system. Densitometry was performed with the Bio-Rad ImageLab 4.0.1 software.

### Immunofluorescent confocal microscopy.

Microscopy and image acquisitions were performed after a 15-min fixation with 4% paraformaldehyde (PFA) followed by blocking with 2% bovine serum albumin (BSA) in PBS for 1 h. HPV L1 was detected using a rabbit polyclonal antibody raised to HPV16 L1 virus-like particles (1:200) (25). This was followed by anti-rabbit IgG Alexa Fluor 555 (AF555) (1:400; Invitrogen, catalog no. A31572) and wheat germ agglutinin (WGA) conjugated to AF488 (1:400; Thermo Fisher, catalog no. W11261). Cells were mounted with ProLong Diamond antifade mountant with DAPI (4′,6-diamidino-2-phenylindole) (Invitrogen, catalog no. P36962) and imaged on a Leica SP8 confocal microscope. Data were collected from three independent experimental replicates wherein three separate fields of view were imaged in three-dimensional (3D) z-stacks for each sample. Fiji ImageJ channel separation was used to isolate the HPV L1 signal for mean fluorescent intensity quantification from the cell and surrounding ECM using WGA as a landmark for cells. Imaging parameters, including laser power, objective, and gain were kept constant in the associated LASX software for all technical and experimental replicates to ensure changes in intensity reflected L1 staining differences.

### Murine vaginal challenge.

The estrus cycles of 6- to 8-week-old female BALB/c mice were synchronized with a subcutaneous injection of 3 mg/mL medroxyprogesterone (Depo-Provera) in PBS (Amphastar Pharmaceuticals) (31). Following synchronization for 5 days, animals were anesthetized with 3% isoflurane (Piramal, catalog no. NDC56794-013-25); nonoxynol-9 (N-9) (Spectrum, catalog no. N1217) at a final 4% concentration in 3% carboxymethylcellulose (CMC) (Sigma, catalog no. C4888) was introduced to the vaginal tract using a positive-displacement pipette. Animals were anesthetized 4 to 6 h after N-9 treatment and vaginally instilled with 1:1 mixed 20 μM PS (final concentration) and 3% CMC (final concentration), totaling 20 μl, for 1 h. Animals were reanesthetized, and thereafter, 1 × 10^9^ VGE of HPV16 PsV was vaginally instilled in 3% CMC by positive-displacement pipette totaling 10 μl so as to not exceed the 30-μl total vaginal volume. Infection was evaluated 48 h post-PsV exposure via instillation of 30 μl of 20 mg/mL luciferin (Xenolight d-luciferin; Perkin Elmer catalog no. 122799). Luciferin was allowed to incubate in the vaginal tract for 5 min prior to a 5-min bioluminescence image acquisition (IVIS Lumina III spectral imager) and analyses with LiveImage software. Regions of interest (ROI) for the lower abdominal area were made uniformly to record luminescence from the vaginal tract of mice. Total luminescence from each ROI was measured in radians (photons/s/cm^2^) and compared across animals and experiments. Groups were normalized to infection in the absence of PS. Animal studies were approved by the UNM IACUC (protocol 17-200423-HSC).

### Infectious focus-forming assay.

RNA *in situ* hybridization (RNA-ISH) for viral E6/E7 mRNAs was used as a quantitative, cell-based focus-forming assay as described previously (47). HaCaT cells were seeded at 5 × 10^4^ cells per well in 8-well chamber slides and exposed to virus stocks. RNAscope RNA-ISH was performed with HPV-HR7 E6/E7 probes (ACD, catalog no. 312351) specific for HPV types 16, 18, 31, 33, 35, 52, and 58. Stained slides were subject to high-definition digital imaging using Aperio Image Scope microscopy (Leica). Positive cells containing ≥2 puncta indicative of HPV E6/E7 mRNA were manually counted as a focus.

### Cellular proliferation assay.

CyQuant cell proliferation assays were performed according to the manufacturer’s instructions (Thermo Fisher, catalog no. C35006). Briefly, HaCaT cells were seeded at 2,500 cells per well in 96-well plates and maintained in 100 μl of complete growth medium to reach exponential growth phase after overnight attachment. Subconfluent cells were treated in triplicate with PS and incubated for 24 h corresponding to infection assays. CyQuant fluorescent DNA intercalation dye (100 μl per well) was added to cells after removal of treatment medium, and plates were incubated at 37°C in 5% CO_2_ for 15 min prior to being transferred to the CLAIROstar Plus fluorescent spectrophotometer (BMG Labtech). Readings were determined at 530 nm, background was determined from empty wells and removed, and averages across triplicates were calculated using CLAIROstar software.

### HA-PS mixing assay.

PS was incubated with high- and low-molecular-weight HA in complete medium at room temperature for 1 h prior to being added to HaCaT cells (high molecular weight, 1,500 to 1,800 kDa; [Sigma-Aldrich, catalog no. 53747]; low molecular weight, 15 to 30 kDa [Sigma-Aldrich, catalog no. 97616]). Concentrations of both forms of HA (sodium salts of HA from Streptococcus equi [Sigma-Aldrich, St. Louis, MO]) were 2 mg/mL in complete medium. PS and HA were mixed in complete medium for 1 h at room temperature with final concentrations after mixture of 3 μM and 1 mg/mL, respectively. Following 1 h of incubation at room temperature, the mixture was added to 1 mL of complete growth medium. Mixtures of HMW or LMW HA and PS and unmixed PS were exposed to 5 × 10^4^ HaCaT cells 1 h prior to infection with HPV16 PsVs and the infection assay.

### HPV internalization assay.

HaCaT cells were seeded at a cell density of 5 × 10^5^ cells/cm^2^ in 6-well plates and allowed to adhere overnight. Thereafter, cells were chilled to 4°C and exposed to 100 VGE/cell of HPV16 PsVs for 1 h at 4°C. After viral attachment, unbound virions were removed by washing, and PS (5.7 μM) was added before cells were transitioned to 37°C for 0, 1, or 4 h to allow for virus internalization. Cells were released from monolayers with 0.25% trypsin–5 mM EDTA for 15 min at 37°C. Trypsin was inactivated with complete HaCaT medium, and the cells were pelleted in a tabletop microcentrifuge. Pelleted cells were washed with ice-cold PBS, and extracellular virus was removed by treatment with ice-cold PBS (pH to 4.5 with 12 N HCl) for 2 min, on ice. Cells were washed three times with PBS and lysed with RIPA buffer. Immunoblots were performed for detection of L1 as described above.

### Statistical analyses.

All analyses were performed in GraphPad Prism 7.02. Infection dose-response curves (luciferase and RT-qPCR results) and proliferation assays were analyzed using one-way analysis of variance (ANOVA) with multiple comparisons. All IC_50_ values were generated using GraphPad inhibitor versus response nonlinear regressions. Densitometry, mean fluorescence intensity, viral pretreatment, viral binding, viral internalization, HA conjugation infection, and *in vivo* luminescence experiments were compared using a two-tailed unpaired Student's *t* test. Welch’s *t* test was used to test for equal means between two sample populations. Significance indicators regardless of test are indicated as follows: n.s., not significant (*P* > 0.05); *, *P* ≤ 0.05; **, *P* ≤ 0.01; ***, *P* ≤ 0.001; ****, *P* ≤ 0.0001.
