# Supplementary material for: Protamine Sulfate Is a Potent Inhibitor of Human Papillomavirus Infection In Vitro and In Vivo
Source: Antimicrob Agents Chemother. 2022 Jan 18;66(1):e01513-21. doi: 10.1128/AAC.01513-21 (PMC8765401; doi:10.1128/AAC.01513-21)
Supplement: Supplemental file 1 — Supplemental material. Download AAC.01513-21-s0001.pdf, PDF file, 0.4 MB [file aac.01513-21-s0001.pdf]

Supplemental Information

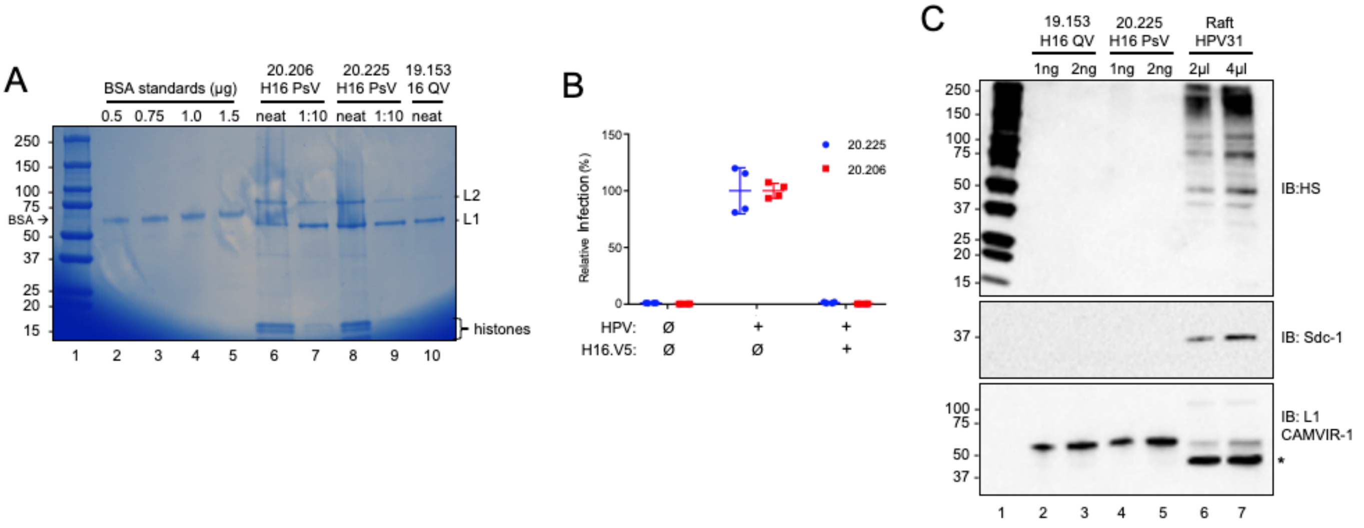

Figure S1. Quality control of HPV physical and infection characteristics. HPV16 PsV and QV stocks isolated by CsCl density gradient centrifugation were assessed for L1 and L2 content (A) and antibody-mediated neutralization (B). (A) Representative SDS-PAGE and Coomassie blue staining of molecular weight standards (kDa, lane 1), bovine serum albumin (BSA) loading controls (lanes 2-5), HPV16 PsV stocks (20.206, lanes 6-7; 20.225, lanes 8-9), and HPV16 QV stock (19.153, lane 10) for assessment of viral capsid components. (B) Representative verification of HPV16 PsV infection including incubation with neutralizing monoclonal antibody H16.V5 (1:1000) for 1h at 37°C prior to exposure to HaCaT cells (n=2 independent infection done duplicate). Infections were quantified by luciferase assay from cell lysates at 24h post PsV exposure with the averages of replicates not exposed to antibody set to 100%. (C) Representative SDS-PAGE and immunoblot (IB) of viral stocks loading ≈1 ng and ≈2 ng of HPV16 QV and PsV and 2 ul and 4 ul of raft-derived HPV31. Immunoblot detection of HS, HSPG core protein Syndecan-1 (sdc-1), and L1. The asterisk (*) marks a ≈42-45 kDa band that may be a breakdown fragment of L1 and/or a nonspecific cellular protein observed in human keratinocytes (1, 2). All other virion stocks were validated previously (3).

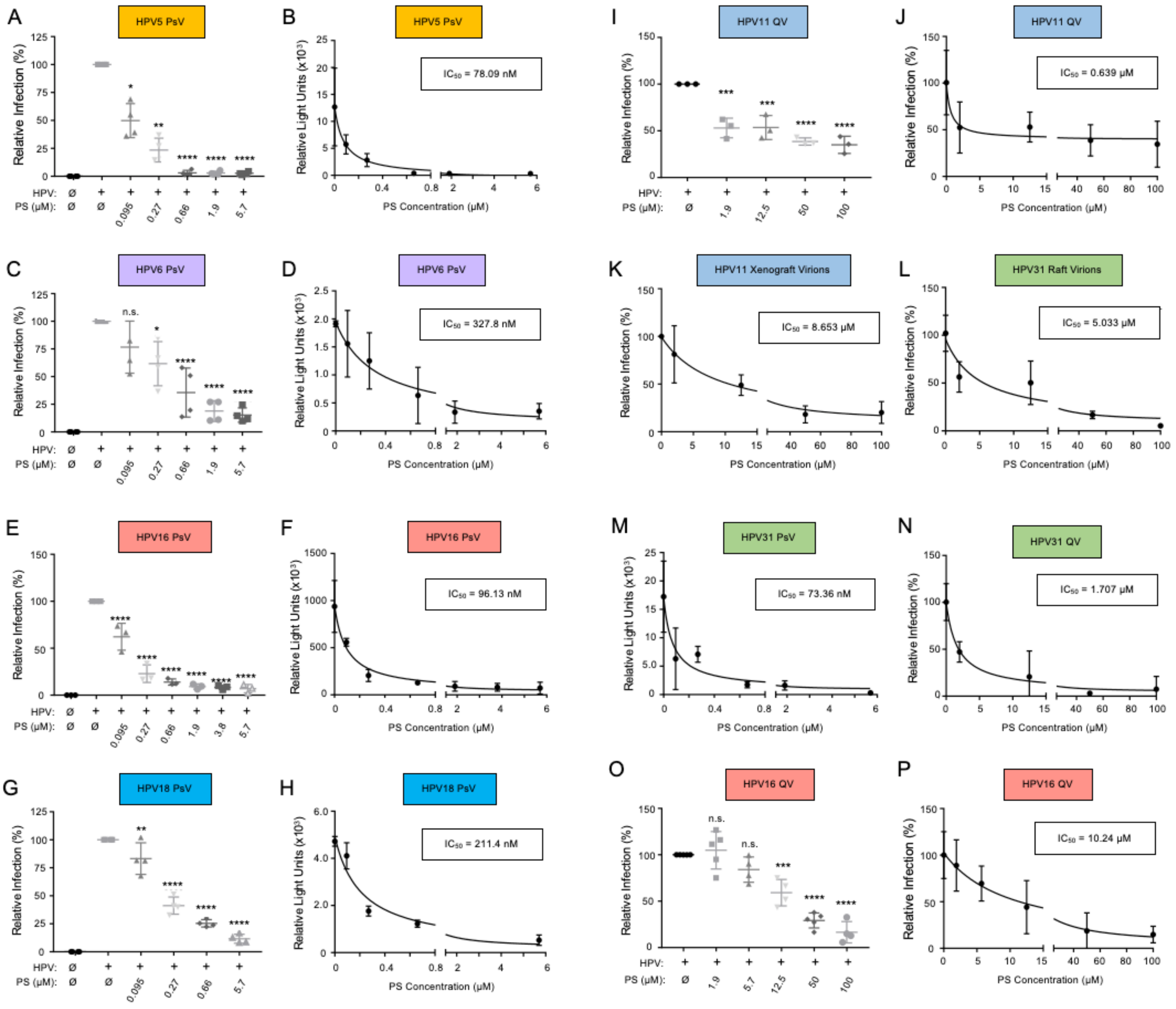

Figure S2. HPV virion infections in protamine sulfate dose *verses* response experiment. HaCaT cells were treated for 1h with increasing concentrations of protamine sulfate (PS) before exposure to PsV or QV at 100 VGE per cell. (A,C,E,G) PS dose *versus* HPV PsV infection response for HPV genotypes and (B,D,F,H) line-of-best-fit non-linear regression based on luciferase units as infection readout as performed in Fig. 1A-B and 2A. (I,O) PS dose *versus* HPV QV infection response for HPV genotypes and (J,P) line-of-best-fit non-linear regression based on RT-qPCR for HPV E1^E4 mRNA levels as performed in Fig. 2B. (K) Line-of-best-fit non-linear regression based on RT-qPCR for HPV11 E1^E4 mRNA levels. (L,M,N) Line-of-best-fit non-linear regression based on RT-qPCR for HPV31 E1^E4 mRNA levels (data from Fig. 2C,D,E).

Table S1. Plasmids used for production of recombinant HPV pseudovirions and quasivirions

Plasmid	Sequences	Function	Source	Reference
**p5-sheLL**	HPV5 L1, L2	L1, L2 capsid protein	Schiller (U.S. NIH)	(4)
**p6-sheLL**	HPV6 L1, L2	L1, L2 capsid protein	Schiller (U.S. NIH)	(4)
**p11L1w**	HPV11 L1	L1 capsid protein (codon optimized)	Schiller (U.S. NIH)	(5)
**p11L2w**	HPV11 L2	L2 capsid protein	Schiller (U.S. NIH)	(6)
**p16 sheLL**	HPV16 L1, L2	L1, L2 capsid proteins	Schiller (U.S. NIH)	(4)
**p18-sheLL**	HPV18 L2	L1, L2 capsid protein	Schiller (U.S. NIH)	(7)
**p31-sheLL**	HPV31 L1, L2	L1, L2 capsid protein	Dürst (DKFZ)	(7)
**pBS-HPV11**	HPV11 genome	Viral genome	de Villiers, zur Hausen (DKFZ)	(8)
**pBS-HPV31**	HPV31 genome	Viral genome	de Villiers, zur Hausen (DKFZ)	(9)
**pBS-HPV16 (114/K)**	HPV16 genome	Viral genome	Dürst (DKFZ)	(10)
**pGL3 control**	luciferase	Viral pseudo-genome	Promega	N/A

Table S2. PCR primers used for viral stock VGE titrations.

Primer Name	Sequence (5' to 3')	HPV Genotype and Target
Luc A	5'-TGC TCC AAC ACC CCA ACA TC-3'	pGL3 Luciferase
Luc B	5'-CCA CAA ACA CAA CTC CTC CGC-3'	pGL3 Luciferase
11qLCRA	5'-TCG GTT GCC CTT TAC ATA CAC-3'	QV11 LCR[1]
11qLCRB	5'-GGT GTG TTG TTG GCA AGA TAC-3'	QV11 LCR
31qLCRA	5'-GGT GTC ACG CCA TAG TAA AAG-3	QV31 LCR
31qLCRB	5'-GGA AAC CAA AAA CCG CAG AAC-3	QV31 LCR
qL1.16A	5'-CCT GAC ACC TCA TTT TAA ATC CAG ATA CAC AG-3'	QV16 LCR
qL1.16B	5'-CAC TAA TGC CCA CAC CTA ATG GCT G-3'	QV16 LCR

[1]Long control region

Table S3. PCR primers and probes used to evaluate HPV infection.

Primer Name	Sequence (5' to 3')	Target
HPV16_837A	5'-CCA TCT GTT CTC AGA AAC CAT-3'	HPV16 E1^E4
HPV16_3402B	5'-GGC CAA GTG CTG CCT AAT-3'	HPV16 E1^E4
HPV16 E1^E4 probe864	5'-6FAM-ATA CTT CGT TGC TGC TGC AGG ATC AGC CAT-MGBNFQ-3'	HPV16 E1^E4
HPV31_E4B	5'-CTT CAC TGG TGC CCA AGG-3'	HPV31 E1^E4
HPV31_E7.4A	5'-GGC TCA TTT GGA ATC GTG TGC-3'	HPV31 E1^E4
HPV31 E1^E4 Probe2	5'-6FAM-CAG TGA CGA AAT ATC CTT TGC TGG ATT GTT-MGBNFQ-3'	HPV31 E1^E4
HPV11_E1.4A	5'-CTG GCG ACC AAA ACC ATA ACA-3'	HPV11 E1^E4
HPV11_E4.4B	5'-GGG TGT ATG TAG TAG GTT CAG CAA TGG A-3'	HPV11 E1^E4
HPV11 E1^E4 Probe1	5'-6FAM-CTT CTC GTA CAG TGC TGA ATC TGC CGC CAT-MGBNFQ-3'	HPV11 E1^E4
B-Actin QA	5'-AGC CTC GCC TTT GCC GA-3'	Cellular ß-actin
B-Actin QB	5'-CTG GTG CCT GGG GCG-3'	Cellular ß-actin
B-Actin QAQB Probe	5'-CCC GCC GCC CGT CCA CAC CCG CC-3'	Cellular ß-actin

References

1. Campos SK, Ozbun MA. 2009. Two highly conserved cysteine residues in HPV16 L2 form an intramolecular disulfide bond and are critical for infectivity in human keratinocytes. PLoS ONE 4:e4463.
2. Cerqueira C, Samperio Ventayol P, Vogeley C, Schelhaas M. 2015. Kallikrein-8 Proteolytically Processes Human Papillomaviruses in the Extracellular Space To Facilitate Entry into Host Cells. J Virol 89:7038-7052.
3. Ozbun MA, Bondu V, Patterson NA, Bennett EC, McKee RG, Waxman AG. 2020. Assessing the Efficacy of Human Papillomavirus Disinfection and the Risk of Transmission from Clinical Lesions. American Journal of Infection Control 48:S3-S4.
4. Buck CB, Thompson CD, Roberts JN, Iler M, Lowy DR, Schiller JT. 2006. Carrageenan Is a Potent Inhibitor of Papillomavirus Infection. PLoS Pathog 2:e69.
5. Mossadegh N, Gissman L, Müller M, Zentgraf H, Alonso A, Tomakidi P. 2004. Codon optimization of the human papillomavirus 11 (HPV 11) L1 gene leads to increased gene expression and formation of virus-like particles in mammalian epithelial cells. Virology 326:57-66.
6. Kieback E, Müller M. 2006. Factors influencing subcellular localization of the human papillomavirus L2 minor structural protein. Virology 345:199-208.
7. Roberts JN, Buck CB, Thompson CD, Kines R, Bernardo M, Choyke PL, Lowy DR, Schiller JT. 2007. Genital transmission of HPV in a mouse model is potentiated by nonoxynol-9 and inhibited by carrageenan. Nat Med 13:857-861.
8. Dartmann K, Schwarz E, Gissmann L, zur Hausen H. 1986. The nucleotide sequence and genome organization of human papilloma virus type 11. Virology 151:124-130.
9. Goldsborough MD, DiSilvestre D, Temple GF, Lorincz AT. 1989. Nucleotide sequence of human papillomavirus type 31: a cervical neoplasia-associated virus. Virology 171:306-311.
10. Kirnbauer R, Taub J, Greenstone H, Roden R, Dürst M, Gissmann L, Lowy DR, Schiller JT. 1993. Efficient self-assembly of human papillomavirus type 16 L1 and L1-L2 into virus-like particles. J Virol 67:6929-6936.
